# Impairments in knowledge of social norms in presymptomatic, prodromal, and symptomatic frontotemporal dementia

**DOI:** 10.1002/dad2.12630

**Published:** 2024-09-03

**Authors:** Liset de Boer, Esther van den Berg, Jackie M. Poos, Willeke Klop, Lucia A. A. Giannini, Julie F. H. De Houwer, Harro Seelaar, Lize C. Jiskoot

**Affiliations:** ^1^ Department of Neurology and Alzheimer Center Erasmus MC University Medical Center Rotterdam the Netherlands; ^2^ Dementia Research Center University College London London UK

**Keywords:** cognition, frontotemporal dementia, neuropsychological assessment, presymptomatic, prodromal

## Abstract

**INTRODUCTION:**

We aimed to assess the knowledge of social norms in patients with behavioral variant frontotemporal dementia (bvFTD) with the Dutch version of the Social Norms Questionnaire (SNQ‐NL).

**METHODS:**

The SNQ‐NL was administered in 34 patients with bvFTD, 20 prodromal mutation carriers, 76 presymptomatic mutation carriers, and 56 controls. Group differences and correlations with other neuropsychological tests and gray matter volume were examined.

**RESULTS:**

Patients with bvFTD had lower total SNQ‐NL scores and more over‐adherence errors than presymptomatic mutation carriers and controls (*P *< 0.001). SNQ‐NL performance correlated with tests for executive functioning and social cognition, and with gray matter volume in bilateral frontal and unilateral temporal regions.

**DISCUSSION:**

The SNQ‐NL can identify impairments in knowledge of social norms in bvFTD, highlighting its significance in clinical diagnosis and upcoming clinical trials. The SNQ‐NL currently fails to differentiate presymptomatic mutation carriers from controls; to this end, larger sample sizes from larger cohorts and longitudinal follow‐up are warranted.

**Highlights:**

- The Dutch version of the Social Norms Questionnaire (SNQ‐NL) is able to detect impairment in social cognition in symptomatic bvFTD patients.
- A trend towards a lower performance in prodromal mutation carriers was found.
- Performance on the SNQ‐NL is related to other measures of social cognition, executive functioning, and language.
- Lower SNQ‐NL performance is related to gray matter volume loss in bilateral frontal and temporal regions.
- The SNQ‐NL provides insight into the underlying cause of deficits in social cognition in bvFTD.

## 1 BACKGROUND

Frontotemporal dementia (FTD) is a young‐onset neurodegenerative disorder that is characterized by disproportional atrophy of the frontal and temporal lobes, causing a progressive decline of behavioral, motor, and cognitive abilities.^1^ The most common clinical presentations constitute the behavioral (bvFTD) and language variants (primary progressive aphasia [PPA]). In ≈ 30%, autosomal dominant mutations in chromosome 9 open reading frame 72 [*C9orf72*], microtubule‐associated protein tau [*MAPT*], and progranulin [*GRN*] are the leading cause of disease.^2^, ^3^ Cognitive decline in genetic forms is increasingly being studied in longitudinal, multicenter cohorts,^3^, ^4^ as we can identify changes in presymptomatic mutation carriers years before overt disease, when pathological damage can still be halted or potentially even reversed.^3^ At present, however, no reliable cognitive biomarkers are available for FTD.

One of the key neuropsychological features of FTD is impairment in social cognition, which refers to the ability to process and interpret social information, such as recognizing and understanding the emotions, intentions, and behaviors of others.^5^, ^6^, ^7^ Social cognition is based on implicit and explicit processes^8^, ^9^ and consists of automatic processing of social information, understanding others, and consciously interpreting the emotional relevance of social information. The most‐studied aspects of social cognition in patients with FTD are emotion recognition (ER; the implicit, rapid, and automatic processing of social stimuli) and Theory of Mind (ToM; the more explicit and controlled processing of social stimuli).^10^ However, social cognition encompasses a broader spectrum of dimensions including the regulation of social behavior, moral decision making, and the understanding and the maintenance of social norms.^10^, ^11^ Several studies have investigated impairments in social normative behavior in FTD spectrum disorders.^8^, ^12^, ^13^, ^14^, ^15^, ^16^ Nevertheless, how these social norms are presented in the presymptomatic and prodromal stages of FTD is still unknown. Identification of impairments in social cognition in these stages could, however, aid in early clinical diagnosis and allow disease monitoring,^17^, ^18^ both important factors with respect to upcoming disease‐modifying trials for genetic FTD. In addition, it could allow the development of psychosocial interventions to strengthen social functioning in patients with (frontotemporal) dementia.^19^, ^20^ Moreover, no consistent data are available yet about the relationships between impairments in social norms and neuropsychological performance and gray matter volume loss in presymptomatic and prodromal stages of FTD, nor about social normative performance in the different FTD mutations.

The version of the Social Norms Questionnaire (SNQ‐NL^11^) used in this study consists of 22 yes–no questions and was previously adapted to Dutch and validated by van den Berg et al.^11^ in patients with bvFTD and Alzheimer's disease (AD) dementia. We investigated whether the SNQ‐NL has the capability to differentiate among patients with bvFTD, prodromal and presymptomatic mutation carriers, and controls. We hypothesized that patients with bvFTD and prodromal mutation carriers exhibit lower total scores on the SNQ‐NL compared to presymptomatic mutation carriers and controls.^8^, ^21^ To test our hypotheses, we compared SNQ‐NL data between groups based on clinical status (i.e., patients with bvFTD, presymptomatic and prodromal mutation carriers, controls). Moreover, to explore the cognitive and neuroimaging correlates of differences in SNQ‐NL performance, we investigated associations with other neuropsychological tests and gray matter (GM) volume loss. Last, we analyzed differences in SNQ‐NL performance within genetic subgroups (*C9orf72, GRN*, and *MAPT*).

## 2 METHODS

### 2.1 Participants

In total, we included 188 participants (age range 26–88). Participants were grouped by means of the Clinical Dementia Rating (CDR) plus Behavior and Language domains from the National Alzheimer's Coordinating Center Frontotemporal Lobar Degeneration (CDR plus NACC‐FTLD^22^) module. Patients with bvFTD (*n* = 34, mutation carriers *n* = 18, sporadic *n* = 16) received a CDR plus NACC‐FTLD global score of ≥ 1, for whom a clinical diagnosis was obtained in a multidisciplinary consensus involving experienced neurologists, neuropsychologists, radiologists, and geriatricians according to the diagnostic consensus criteria for probable or definite bvFTD.^23^ Patients with other clinical syndromes within the FTD spectrum (e.g., PPA, corticobasal syndrome, progressive supranuclear palsy) and mixed types were excluded from our study to ascertain homogeneity of the sample and to minimize the influence of speech and/or language disorders on SNQ‐NL performance. Prodromal mutation carriers (*n* = 20, mutation carriers *n* = 14, sporadic *n* = 6) had a CDR plus NACC‐FTLD global score of 0.5. Presymptomatic mutation carriers (*n* = 76; *C9orf72*; *n *= 53, *GRN*; *n *= 35, *MAPT*; *n *= 19 or TAR‐DNA‐binding protein [TARDBP; *n *= 3]) were not fulfilling clinical diagnostic criteria for bvFTD^23^ and had a CDR plus NACC‐FTLD global score of 0.^22^ Last, we included 56 cognitively healthy controls, consisting of healthy first‐degree (i.e., mutation‐negative) family members of patients with genetic FTD.

### 2.2 Procedure

Presymptomatic mutation carriers and controls were enrolled between January 2010 and June 2023 in the FTD Risk Cohort (FTD‐RisC), a longitudinal study in which 50% at‐risk participants for one of the FTD gene mutations are being followed annually.^4^ Prodromal and symptomatic mutation carriers were either enrolled in the FTD‐RisC study and/or were visiting the outpatient memory clinic of the Alzheimer and FTD Expertise Centre of the Erasmus University Medical Center (Rotterdam, the Netherlands). All participants underwent a standardized clinical interview and neurological and neuropsychological assessment. Most participants underwent laboratory testing (lumbar puncture and/or blood sampling) and structural magnetic resonance (MR) imaging of the brain. To measure global cognitive and frontal–executive dysfunction, the Mini‐Mental State Examination (MMSE^24^) and Frontal Assessment Battery (FAB^25^), respectively, were administered.

### 2.3 Consent statement

Ethical approval was obtained from the local ethics committee (MEC‐2009‐409) for the FTD‐RisC study and the local biobank study that included participants enrolled from the outpatient memory clinic (MEC‐2016‐069). All participants gave written informed consent.

### 2.4 SNQ‐NL

The SNQ‐NL was administered to all participants as part of the routine neuropsychological assessment. The SNQ‐NL consists of standardized instruction and 22 yes–no questions.^11^ Examples of questions are “Would it be socially acceptable to ask a coworker's age? (yes)” or “Would it be socially acceptable to spit on the floor? (no).” A total score was calculated by adding the number of right answers (0 to 22). Two types of errors were scored in addition to the total score: break and over‐adherence errors. A break error refers to endorsement of a socially inappropriate behavior (e.g., eating pasta with your fingers) as appropriate; an over‐adherence error refers to endorsement of a socially appropriate behavior (e.g., wearing the same shirt twice in 2 weeks) as inappropriate.^26^ The break error score was the sum of all break errors made (0 to 12, a higher score reflects worse performance), the over‐adherence error score was the sum of all over‐adherence errors (0 to 10, a higher score reflects worse performance). Exclusion criteria for the study were having one or more missing items on the SNQ‐NL. According to Knopman and Kukull,^27^ a yes/no ratio score of < 0.3 or > 5 (the amount of “yes” answers divided by the amount of “no” answers) could indicate bias unrelated to the content of the items. In our study, 16 participants had a yes/no ratio score of < 0.3. We decided to include these participants to explore in which CDR plus NACC‐FTLD groups these ratio scores were most common to shed light on their clinical relevance and provide an understanding of the test's outcomes.

### 2.5 Neuropsychological correlates

We explored the cognitive associations of the SNQ‐NL by analyzing associations with other neuropsychological tests from the standardized neuropsychological test battery. We chose the neuropsychological tests based on the correlations found by van den Berg et al.^11^ As a measure of social cognition, the Emotion Recognition Task (ERT^28^) was included. The Trail Making Test (TMT) Part A^29^ was included as a measure of information processing speed. TMT Part B, and category and letter fluency tests,^30^ were included as measures of executive functioning. Last, the 60‐item Boston Naming Test (BNT60) was included to measure language (naming) abilities.^31^

**RESEARCH IN CONTEXT**

1. **Systematic review**: The authors reviewed the literature using traditional (e.g., PubMed) sources. While knowledge of social norms has not been studied systematically in different clinical stages in (genetic) frontotemporal dementia (FTD), there have been several publications describing neuropsychological test results, including social cognitive tests, in (genetic) FTD. Relevant citations are cited.
2. **Interpretation**: Our findings demonstrate a deficit in knowledge of social norms in the behavioral variant of FTD (bvFTD), even in a subset of prodromal individuals. These results are consistent with previous studies showing deficits in social cognition in patients with bvFTD.
3. **Future directions**: Results from this study provide new insights and guidance for future research, such as investigating longitudinal trajectories of the SNQ‐NL in genetic FTD to examine its significance in diagnostic processes and upcoming clinical trials.

### 2.6 Structural brain imaging and voxel‐based morphometry

Images were acquired on a 3T MRI scanner (Philips Achieva). Participants from the FTD‐RisC study underwent MR imaging during the same visit as the neuropsychological assessment. Participants from the outpatient memory clinic underwent MR imaging in a period of ± 3 months before or after SNQ‐NL administration. All scans underwent extensive visual quality checks and images with large artifacts and/or incidental brain abnormalities unrelated to bvFTD were excluded from further analysis (*n *= 13). In total, 104 volumetric T1 scans were processed using the VBM Toolbox in Statistical Parametric Mapping 12 (SPM12; www.fil.ion.ucl.ac.uk/spm, version 7771, running in Matlab R2021b [Mathworks]). Images were realigned to correct for motion artifacts and then skull extracted and segmented to obtain the GM, white matter (WM), and cerebrospinal fluid (CSF) volumes. GM segmentations were transformed into Montreal Neurological Institute (MNI) space, modulated, and smoothed using a Gaussian kernel filter of 6 mm. A GM mask was applied by using the Masking toolbox.^32^ A partial correlation analysis was conducted between the SNQ‐NL scores and the GM volumes by means of multiple regressions.

### 2.7 Statistical analysis

Statistical analyses were performed using SPSS statistics version 28.0.1.0 (IBM Corp.) and GraphPad Prism 5. The significance level was set at *P *< 0.05 (two‐tailed) across all comparisons and we implemented corrections for multiple testing. To compare demographic data between groups, we used general linear models with Tukey post hoc tests. We analyzed differences in sex using chi‐square tests. The distribution of all SNQ‐NL scores and the TMT‐A and ‐B and BNT60 deviated from a normal distribution, hence non‐parametric counterparts of the abovementioned tests were used. Quade non‐parametric analyses of covariance (ANCOVAs) were conducted to compare the mean differences in SNQ‐NL performance (total, break, and over‐adherence error scores) across participant groups (*n *= 188), while controlling for the effects of age, sex, and years of education. A chi‐square test was performed to analyze group differences in the number of ratio scores of < 0.3. To explore genetic effects, we pooled all mutation carriers (*n *= 110), irrespective of their clinical status due to small sample sizes, and divided them by genetic group (*C9orf72, GRN*, and *MAPT*). A Quade non‐parametric ANCOVA was conducted to compare the mean differences in SNQ‐NL performance (total, break, and over‐adherence error scores) across the genetic groups while controlling for the effects of age, sex, years of education, and CDR plus NACC‐FTLD global score. *TARDP* mutation carriers were excluded from this analysis due to the small sample size of this group. A partial correlation analysis per CDR plus NACC‐FTLD global score was performed with age, sex, and years of education as covariates to analyze the relationships with SNQ‐NL performance and the other neuropsychological measures. We used bootstrapping (*n *= 1000) to account for assumption relaxation and to estimate confidence intervals. The relationship of performance on each SNQ‐NL score with GM volume was explored by using multiple regression models in the VBM analysis. Age, sex, years of education, and Total Intracranial Volume (TIV) were included as covariates. All comparisons were corrected for a family‐wise error rate of 0.05.

## 3 RESULTS

### 3.1 Demographic and clinical data

Demographic, clinical, and neuropsychological data are presented in Table 1. Patients with bvFTD and prodromal mutation carriers were older than presymptomatic mutation carriers and controls (*F*[3,184] = 13.119, *P *< 0.001–0.034). There was a significant difference between groups in the years of education (*F*[3,184) = 5.050, *P* = 0.004–0.016). Controls and presymptomatic mutation carriers had a higher level of education compared to patients with bvFTD (*F*[3,149] = 3.622, *P *< 0.001–0.024). There were no significant differences in sex between groups (χ^2^[3] = 4.092, *P *> 0.05). Patients with bvFTD and prodromal mutation carriers scored significantly lower on the MMSE compared to presymptomatic mutation carriers and controls (F[3,175] = 24.495, *P *< 0.001). Additionally, patients with bvFTD and prodromal mutation carriers scored significantly lower on the FAB compared to presymptomatic mutation carriers and controls, and patients with bvFTD scored significantly lower than prodromal mutation carriers (*F*[3,158] = 20.557, *P *< 0.001–0.007).

**TABLE 1:** Demographic and clinical data in the total sample (*n *= 188).

	Patients	Prodromal mutation carriers	Presymptomatic mutation carriers		
	CDR plus NACC‐FTLD global score ≥1 (*n *= 34)	CDR plus NACC‐FTLD global score 0.5 (*n *= 20)	CDR plus NACC‐FTLD global score 0 (*n *= 78)	Controls *n *= 51	Statistical differences
Sex	14 F/20 M	9 F/11 M	44 F/34 M	34 F/22 M	*Sym = pro = pre = con*
Age	62.7 ± 9.9 (range 41–77)	63.8 ± 12.3 (range 44–88)	49.5 ± 32.2 (range 26–80)	55.1 ± 12.1 (range 30–82)	*Sym = pro > con = pre*
Genetic status	6 *C9orf72* 5 *GRN* 5 *MAPT* 2 *TARDP* 16 sporadic	6 *C9orf72* 5 *GRN* 3 *MAPT* 6 sporadic	41 *C9orf72* 25 *GRN* 11 *MAPT* 1 *TARDP*	N/A	
Years of education	11.4 ± 9.9	11.9 ± 3.5	13.6 ± 3.0	13.5 ± 3.1	*Sym < pre = con*
SNQ‐NL total score (/22)	15.0 ± 3.7	17.6 ± 2.7	19.0 ± 1.5	19.0 ± 1.5	*Sym < pro = pre = con*
SNQ‐NL break error score (/12)	2.0 ± 2.6	1.2 ± 1.1	1.2 ± 1.5	1.0 ± 0.9	*Sym = pro = pre = con*
SNQ‐NL over‐adherence error score (/10)	5.1 ± 2.5	3.3 ± 2.8	1.9 ± 1.3	2.1 ± 1.5	*Sym > pro = pre = con*
MMSE (/30)	25.8 ± 4.1	26.7 ± 2.8	29.2 ± 1.3	29.2 ± 0.9	*Sym = pro < pre = con*
FAB (/18)	13.5 ± 3.8	15.7 ± 3.0	17.3 ± 1.3	17.3 ± 1.3	*Sym < pro < pre = con*
ERT (/100)	36.1 ± 10.2	48.5 ± 10.1	61.6 ± 8.9	60.3 ± 9.9	*Sym < pro < pre = con*
TMT‐A, seconds (/300)	58.8 ± 41.0	38.6 ± 22.0	28.0 ± 15.0	27.3 ± 17.6	*Sym = pro > pre = con*
TMT‐B, seconds (/300)	165.4 ± 94.6	118.3 ± 82.2	63.0 ± 33.9	66.7 ± 43.2	*Sym = pro > pre = con*
Category fluency (animals, total score)	13.8 ± 6.9	20.1 ± 7.8	24.5 ± 5.2	26.2 ± 6.8	*Sym = pro < pre = con*
Letter fluency (3 letters, total score)	18.7 ± 14.1	34.8 ± 14.9	41.9 ± 14.8	46.4 ± 11.9	*Sym < pro = pre = con*
BNT60 (/60)	42.4 ± 10.4	48.1 ± 11.2	54.9 ± 4.6	55.6 ± 4.6	*Sym < pro < pre = con*

*Note*. Data are presented as mean ± standard deviation.

Abbreviations: BNT60, Boston Naming Test 60 items; *C9orf72*, chromosome 9 open reading frame 72; CDR, Clinical Dementia Rating scale; con, controls; ERT, Emotion Recognition Task; F, female; FAB, Frontal Assessment Battery; *GRN*, progranulin; M, male; *MAPT*, microtubule‐associated protein tau; MMSE, Mini‐Mental State Examination; NACC‐FTLD, National Alzheimer's Coordinating Center Frontotemporal Lobar Degeneration module; pre, presymptomatic; pro, prodromal; SNQ‐NL, Social Norms Questionnaire—Dutch version; sym, symptomatic; *TARDP*, *TAR‐DNA‐binding protein*; TMT‐A, Trail Making Test, Part A; TMT‐B, Trail Making Test, Part B.

### 3.2 SNQ‐NL performance between groups

**FIGURE 1:**
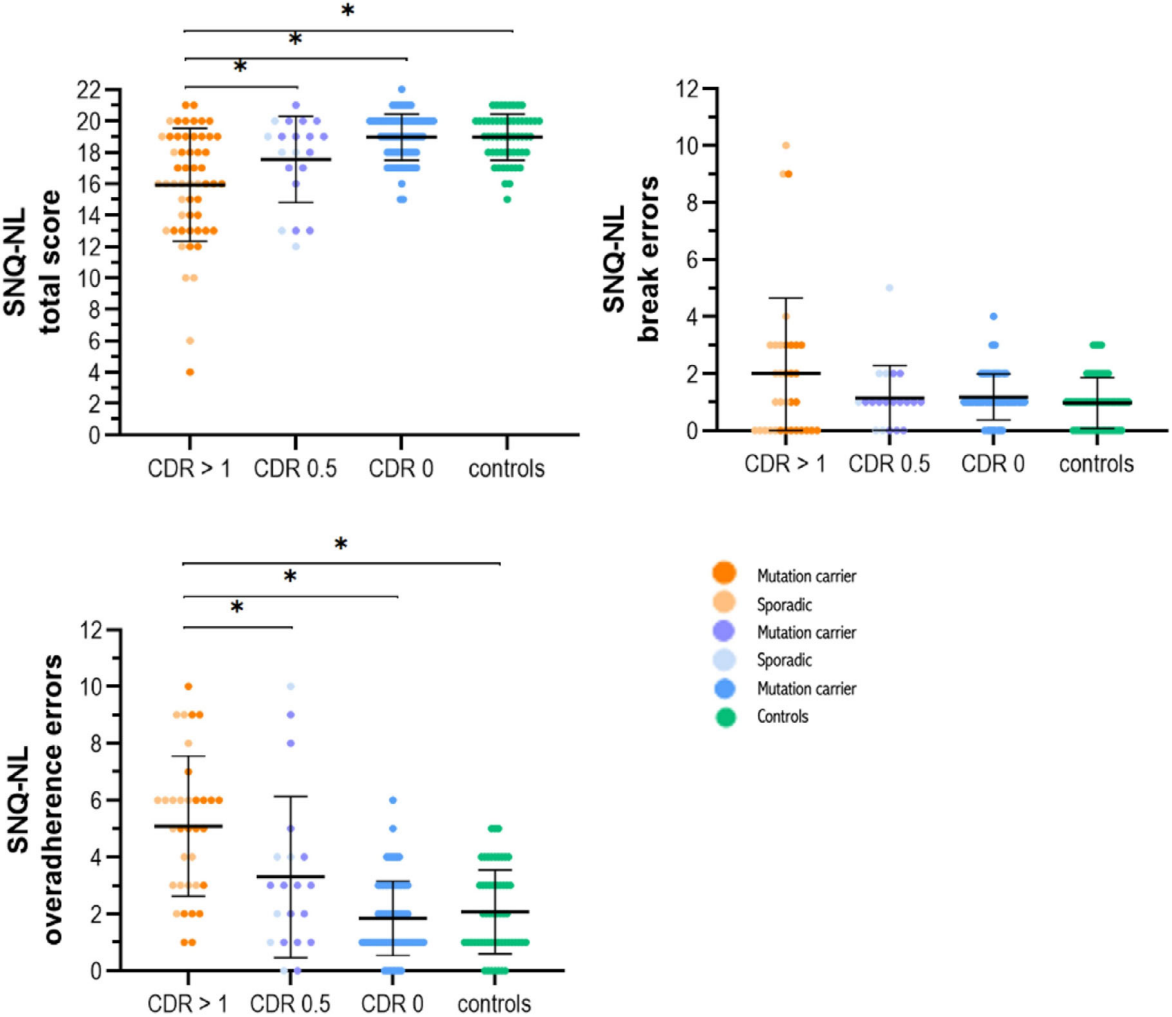
Mean and standard deviation of SNQ‐NL performance per CDR plus NACC‐FTLD group. *P < 0.05. CDR, Clinical Dementia Rating scale; NACC‐FTLD, National Alzheimer's Coordinating Center Frontotemporal Lobar Degeneration module; SNQ‐NL, Dutch version of the Social Norms Questionnaire

Patients with bvFTD had lower SNQ‐NL total scores (mean = 15) compared to prodromal and presymptomatic mutation carriers, and controls (*F*[3,184] = 8.248, *P *< 0.001; Figure 1). Prodromal mutation carriers (mean = 17.6) had lower total SNQ‐NL scores compared to presymptomatic mutation carriers and controls (mean = 19.0 and 19.0, respectively), although not significantly (*P *> 0.05; Figure 1). There are no differences between groups concerning the SNQ‐NL break errors (*P *= 0.366). Patients with bvFTD made more over‐adherence errors compared to prodromal and presymptomatic mutation carriers, and controls (*F*[3,184] = 8.981, *P *< 0.001). Patients with bvFTD had more ratio scores of < 0.3 compared to prodromal and presymptomatic mutation carriers, and controls (χ^2^[3] = 42.914, *P *< 0.001). *MAPT* mutation carriers made more over‐adherence errors compared to the other genetic groups, but after correcting for CDR plus NACC‐FTLD global score, differences were not statistically significant (Figure S1 in supporting information). No significant differences in SNQ‐NL total score or break errors were found between the genetic groups.

### 3.3 Neuropsychological correlates

Correlation coefficients per CDR plus NACC‐FTLD score for each SNQ‐NL score with other neuropsychological tests are presented in Table 2. In the control group, a higher SNQ‐NL total score and fewer break errors were associated with better performance on the ERT (*r* = −0.35‐0.32, *P *= 0.01–0.03). In the prodromal group, a higher SNQ‐NL total score and fewer over‐adherence errors correlated with better performance on TMT‐B (*r *= −0.84–0.70, *P *< 0.01). In patients, a higher SNQ‐NL total score and fewer over‐adherence errors were associated with better performance on TMT‐A (*r* = −0.64–0.60, *P *= 0.01–0.02) and TMT‐B (r = −0.55–0.66, *P *= 0.03–0.01). Fewer over‐adherence errors were associated with better performance on category fluency (*r* = −0.79, *P *< 0.01). Moreover, higher performance on the SNQ‐NL shows moderate correlations with the ERT in prodromal mutation carriers and patients; however, this was not significant (*P *= 0.06 and *P *= 0.11, respectively). Correlation coefficients in other defined clinical groups (controls–presymptomatic, presymptomatic–prodromal, prodromal–patients) are provided in Table S1 in supporting information.

**TABLE 2:** Correlation coefficients per clinical group (based on CDR plus NACC‐FTLD score) between SNQ‐NL scores and other neuropsychological tests.

	ERT	TMT‐A	TMT‐B	Letter fluency	Category fluency	BNT60
**Controls**						
SNQ‐NL total score	0.32*	0.11	−0.04	0.20	−0.15	0.09
SNQ‐NL break error score	−0.35*	−0.07	0.08	−0.21	−0.01	−0.04
SNQ‐NL over‐adherence error score	−0.10	−0.07	−0.02	−0.07	0.18	−0.07
**Presymptomatic mutation carriers**						
SNQ‐NL total score	−0.02	0.01	−0.17	0.22	0.19	0.05
SNQ‐NL break error score	0.08	−0.12	0.06	−0.08	−0.09	0.03
SNQ‐NL over‐adherence error score	−0.11	0.05	0.18	−0.16	−0.15	−0.10
**Prodromal mutation carriers**						
SNQ‐NL total score	0.48	0.03	−0.84*	−0.15	0.11	0.01
SNQ‐NL break error score	−0.26	−0.04	0.59*	0.22	0.10	0.29
SNQ‐NL over‐adherence error score	−0.46	−0.01	0.70*	0.05	−0.21	−0.20
**Patients**						
SNQ‐NL total score	0.35	−0.64*	−0.55*	0.30	0.51	0.24
SNQ‐NL break error score	−0.02	0.43	0.20	−0.01	0.03	−0.39
SNQ‐NL over‐adherence error score	−0.50	0.60*	0.66*	0.43	−0.79*	−0.04

*Notes*: * = Correlation is significant at the 0.05 level (2‐tailed).

Abbreviations: BNT60, 60‐item Boston Naming Test; CDR, Clinical Dementia Rating scale, ERT, Emotion Recognition Task; SNQ‐NL, Social Norm Questionnaire, Dutch version; TMT‐A, Trail Making Test, Part A; TMT‐B, Trail Making Test, Part B.

### 3.4 Neuroanatomical correlates

**FIGURE 2:**
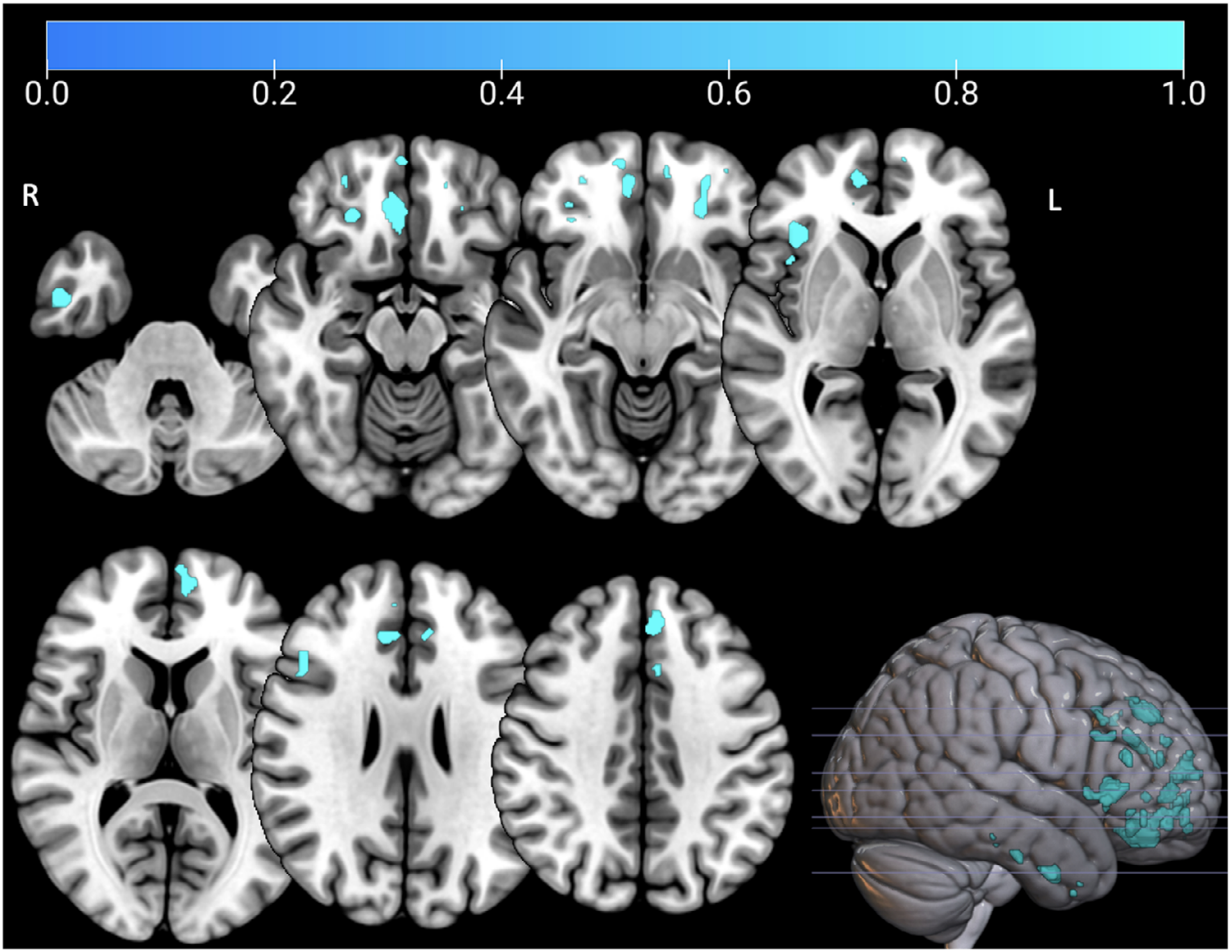
Neuroanatomical correlates of performance on the Dutch version of the Social Norms Questionnaire total score. Results are shown on a study‐specific T1‐weighted magnetic resonance imaging template in Montreal Neurological Institute space. Results are corrected for family‐wise error (*P* < 0.05).

T1 scans of patients with bvFTD, and prodromal and presymptomatic mutation carriers (total *n *= 104) were analyzed to identify neuroanatomical correlates of SNQ‐NL performance (Figure 2). VBM analyses showed a significant association between bilateral frontal (i.e., ventromedial prefrontal cortex, orbitofrontal cortex, anterior insula) and temporal regions (i.e., inferior temporal gyrus) and the SNQ‐NL total score (Figure 2, Table S2 in supporting information): the lower the SNQ‐NL performance, the lower the GM volume of abovementioned frontotemporal regions.

## 4 DISCUSSION

This study demonstrated the presence of impairment in knowledge of social norms in patients with bvFTD, and a negative trend in performance in a subgroup of prodromal mutation carriers. The difference in SNQ‐NL total performance was mostly driven by the number of over‐adherence errors. Performance on the SNQ‐NL was significantly, though moderately, correlated with performance on the ERT in controls and TMT‐A, TMT‐B, and category fluency in affected participants. In addition, lower SNQ‐NL performance was related to lower GM volumes of bilateral frontal (ventromedial prefrontal cortex, orbitofrontal cortex, anterior insula) and temporal (inferior temporal gyrus) regions. Taken together, these findings indicate that the SNQ‐NL is a sensitive test for discriminating patients with bvFTD from presymptomatic mutation carriers and healthy controls. Lower performance on the SNQ‐NL might indicate conversion in prodromal bvFTD.

The findings of the current study are in line with previous work demonstrating impairments in the knowledge of social norms^8^, ^12^, ^13^, ^14^, ^15^, ^16^ and other modalities of social cognition in patients with bvFTD.^33^, ^34^, ^35^ In addition, we found moderate correlations between the SNQ‐NL total and over‐adherence error scores and measures for social cognition (controls) and executive functioning (prodromal mutation carriers and patients). It can be hypothesized that the SNQ‐NL is associated with these measures as it relies both on social cognition (knowledge of social norms) and executive functioning (evaluating social norms in different contexts). These findings are partially in line with the study of van den Berg et al.,^11^ finding moderate correlations between the SNQ‐NL and the ERT and verbal fluency. An explanation of the lack of significant correlations with over‐adherence errors in the previous study could be the inclusion of patients with disorders other than bvFTD (i.e., AD and psychiatric disorders), while the current study solely focused on different clinical stages of FTD. In patients with bvFTD, we expect stronger associations with tests for mental flexibility, as this domain is—next to social cognition—often more impaired in bvFTD compared to AD and psychiatric disorders, and more strongly related to over‐adherence errors. In addition, it is conceivable that there are differences in the nature and extent of impairments in social cognition in bvFTD compared to AD, which was already found for ER and ToM.^36^, ^37^, ^38^

Regarding brain imaging analyses, we found associations with the SNQ‐NL total score and frontotemporal GM regions, specifically the medial prefrontal cortex (mPFC) and orbitofrontal cortex (OFC) as they are known to be involved in complex social behavior and emotion–cognition interactions,^39^ and are often the first regions to become affected in bvFTD.^40^ Moreover, the temporal lobes are involved in social semantic knowledge and higher social function through connections with the mPFC, OFC, and amygdala.^14^, ^41^ No associations were found with subcortical structures, which is surprising as previous studies reported associations between social cognitive measures (i.e., ToM and social communication) and GM volume of primarily the thalamus,^42^, ^43^ but also the hippocampus and amygdala.^43^ Larger sample sizes are needed to further explore the neuroimaging correlates of SNQ‐NL performance, given subcortical atrophy (most specifically of the thalamus) is a consistent finding in genetic subtypes of FTD^44^, ^45^ and is found to be associated with facial ER in *GRN* mutation carriers.^35^

In addition, patients with bvFTD made more over‐adherence errors compared to prodromal and presymptomatic mutation carriers and controls. As a result, patients with bvFTD had more ratio scores of < 0.3. In previous work, SNQ over‐adherence errors were described as non‐specific and to be associated with anxiety, inattention, or a global decrease in cognitive functioning.^8^, ^46^ However, in this study, over‐adherence errors were associated with worse executive functioning. It is conceivable that patients with bvFTD have knowledge of social norms, but do not have the cognitive flexibility and/or language capabilities to evaluate/apply the social norm in different social contexts. For example, eating with your hands is often not acceptable (e.g., when eating pasta), but in some situations (e.g., when eating fries) it is. These results correspond with findings from a previous study in which patients with bvFTD demonstrated impairments in the evaluation of social contexts.^47^ Social norm violations, as reflected in break errors, could be associated with disinhibition, leading to increased social rule‐breaking in bvFTD.^11^ Although patients with bvFTD usually present more behavioral disinhibition, we did not find more break errors in our patient sample. A possible explanation could be the lack of sensitivity in the detection of impaired social behavior and rule‐breaking by traditional neuropsychological measures and questionnaires, as proposed by Panchal et al.^14^

Interestingly, *MAPT* mutation carriers performed slightly worse on the SNQ‐NL and made more over‐adherence errors compared to the other genetic groups; however, after correcting for global cognitive impairment this finding was not significant. Nevertheless, temporal involvement in *MAPT* mutation carriers may play a role in lower performance, as the temporal lobes are often affected in *MAPT*‐related FTD, resulting in loss of memory, language, and semantic capabilities.^2^, ^48^, ^49^ The SNQ‐NL might require these capabilities to adequately identify the social norms and to accurately respond to them. In addition, *MAPT* mutation carriers are known for their rapid cognitive decline once they become symptomatic,^2^ probably causing lower performance on neuropsychological tests compared to patients with a *GRN* or *C9orf72* mutation in their early symptomatic stages.

A strength of this study is the use of a cohort of well‐defined patients with bvFTD and the use of a matched control group of mutation‐negative family members. Furthermore, the mutation carriers are divided into presymptomatic, prodromal, and symptomatic based on CDR plus NACC FTD scores, whereas other studies investigated the differences between presymptomatic and symptomatic mutation carriers only.^34^, ^35^ Nonetheless, the data of the current study should be interpreted in light of some limitations. One limitation is the relatively small sample sizes once stratified. Future studies should aim for larger study groups (e.g., by including [genetic] bvFTD patients from other countries) as well as investigate longitudinal data to create better profiles of social cognition. Because of these small sample sizes, we decided to pool our genetic groups (including all CDR plus NACC‐FTLD scores per genetic group), which has increased statistical power but could have obscured gene‐specific effects. Another limitation lies in the construction of the SNQ‐NL itself; it might not be sensitive enough to identify impairment in all stages of bvFTD (i.e., presymptomatic and prodromal) as it measures a modality of social cognition that also requires executive functioning, memory, and language with yes–no answers, not allowing for more nuanced responses. This limitation could have caused the absence of potential (subtle) differences in performance between genetic groups. Future studies should look for more implicit, innovative tests to measure social norms identification and knowledge, for example by using social situations in virtual reality. Furthermore, the CDR plus NACC‐FTLD scoring comes with some limitations. The assessment of motoric and neuropsychiatric symptoms is currently not included in this staging method, causing a potential possibility that some patients with such symptoms are miscategorized as a score of 0.5 or ≥ 1 while these individuals are in fact in a more advanced disease stage (this applies especially to *C9orf72* repeat expansion carriers). This could explain the higher variability in SNQ‐NL scores that was observed in patients and in the prodromal group. Last, in the presymptomatic group, there is more variability in age. Young mutation carriers are also included, while we predict that the development of initial symptoms will be later (> 10 years) compared to the older mutation carriers. Future studies with larger sample sizes could divide the presymptomatic group into early‐presymptomatic and late‐presymptomatic groups to explore this further.^35^ In the current study, we decided against that (and added age as a covariate instead) due to the resulting small sample size in each group and the need for sufficient statistical power.

To summarize, we demonstrated significant impairment in the identification of social norms in patients with bvFTD and a trend toward a lower performance in prodromal mutation carriers, highlighting the SNQ's significance in the diagnostic work‐up of memory clinics and upcoming clinical trials for FTD. The SNQ‐NL currently fails to differentiate presymptomatic mutation carriers from controls and to this end, larger sample sizes from larger (international) cohorts and longitudinal follow‐up that allows comparisons between early‐presymptomatic and late‐presymptomatic cases, and investigation of differential diagnostic performance to other neuropsychological tests, are still warranted.

## CONFLICT OF INTEREST STATEMENT

The authors declare no conflicts of interest.

## Supporting information

Supplementary figure S1

Supplementary Information

Supplementary Information

Supplementary Information

Supplementary Information
