# Supplementary figures and images for: Impairments in knowledge of social norms in presymptomatic, prodromal, and symptomatic frontotemporal dementia
Source: Alzheimers Dement (Amst). 2024 Sep 3;16(3):e12630. doi: 10.1002/dad2.12630 (PMC11369490; doi:10.1002/dad2.12630)

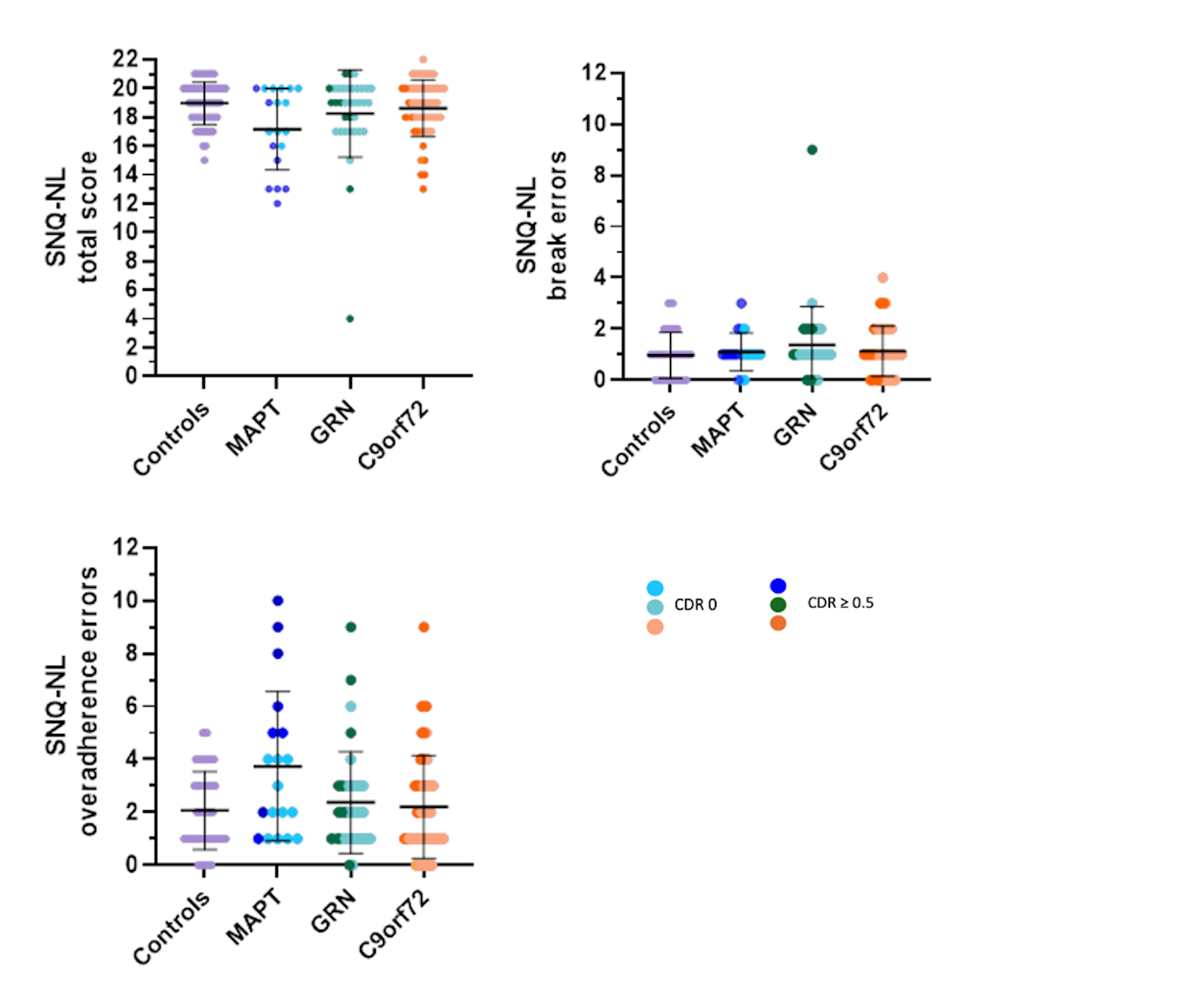

Supplement: Supplementary file 2 — Supplementary Information [file DAD2-16-e12630-s001.jpg]
