# Supplementary material for: Impairments in knowledge of social norms in presymptomatic, prodromal, and symptomatic frontotemporal dementia
Source: Alzheimers Dement (Amst). 2024 Sep 3;16(3):e12630. doi: 10.1002/dad2.12630 (PMC11369490; doi:10.1002/dad2.12630)
Supplement: Supplementary file 3 — Supplementary Information [file DAD2-16-e12630-s003.docx]

**Supplementary table 1** Correlation coefficients per group (controls-presymptomatic mutation carriers, presymptomatic mutation carriers-prodromal mutation carriers & prodromal mutation carriers-patients) between SNQ-NL scores and other neuropsychological tests. Note: * = Correlation is significant at the 0.05 level (2-tailed). Abbreviations: CDR, Clinical Dementia Rating scale, SNQ-NL, Social Norm Questionnaire, Dutch version; ERT, Emotion Recognition Test; TMT, Trail Making Test; BNT60, 60-item Boston Naming Test

|  | **ERT** | **TMT A** | **TMT B** | **Letter**  **fluency** | **Category**  **fluency** | **BNT60** |
| --- | --- | --- | --- | --- | --- | --- |
| **Controls & presymptomatic mutation carriers** |  |  |  |  |  |  |
| SNQ-NL  total score | 0.12 | 0.05 | -0.10 | 0.21* | 0.04 | 0.06 |
| SNQ-NL  break error score | -0.11 | -0.08 | 0.07 | -0.15 | -0.07 | 0.01 |
| SNQ-NL overadherence  error score | -0.05 | -0.02 | 0.07 | -0.11 | 0.01 | -0.09 |
| **Presymptomatic mutaton carriers & prodromal mutation carriers** |  |  |  |  |  |  |
| SNQ-NL  total score | 0.08 | 0.01 | -0.33* | 0.15 | 0.15 | 0.06 |
| SNQ-NL  break error score | -0.06 | -0.07 | 0.30* | -0.07 | -0.04 | 0.00 |
| SNQ-NL overadherence  error score | -0.04 | 0.03 | 0.21 | -0.09 | -0.15 | -0.09 |
| **Prodromal mutation carriers & patients** |  |  |  |  |  |  |
| SNQ-NL  total score | 0.49* | -0.53* | -0.58* | 0.30 | 0.48* | 0.32 |
| SNQ-NL  break error score | -0.07 | 0.35 | 0.13 | 0.03 | 0.04 | -0.23 |
| SNQ-NL overadherence  error score | -0.57* | 0.42* | 0.64* | -0.40* | -0.62* | -0.23 |
