# Supplementary material for: Impairments in knowledge of social norms in presymptomatic, prodromal, and symptomatic frontotemporal dementia
Source: Alzheimers Dement (Amst). 2024 Sep 3;16(3):e12630. doi: 10.1002/dad2.12630 (PMC11369490; doi:10.1002/dad2.12630)
Supplement: Supplementary file 4 — Supplementary Information [file DAD2-16-e12630-s002.docx]

**Supplementary table 2** Significant anatomical regions and MNI coordinates of the VBM analysis. Only regions with a cluster higher than 10 are presented in the table. Abbreviations: MNI, Montreal Neurological Institute; T, height threshold; P_FWE_, family wise corrected p-value.

| **Anatomical region** | **MNI coordinates (X, Y, Z)** | **Cluster** | **T** | **P_FWE-corrected_** |
| --- | --- | --- | --- | --- |
| **Insula R** | 39, 22, -2 | 254 | 6.04 | 0.000 |
| **Frontal Medial Orbital L** | -10, 57, 12 | 210 | 6.05 | 0.000 |
| **Frontal Medial Orbital L** | -12, 54, -8 | 102 | 5.53 | 0.001 |
| **Frontal Medial Orbital L** | 2, 58, -15 | 22 | 5.32 | 0.011 |
| **Frontal Medial Orbital R** | 9, 50, 2 | 92 | 5.61 | 0.001 |
| **Frontal Medial Orbital R** | 28, 50, -12 | 38 | 5.35 | 0.006 |
| **Frontal Superior Medial L** | -3, 38, 36 | 205 | 5.74 | 0.000 |
| **Frontal Inferior Orbital L** | -26, 38, -10 | 123 | 5.96 | 0.000 |
| **Frontal Inferior Orbital R** | 24, 34, -15 | 61 | 5.24 | 0.003 |
| **Frontal Inferior Orbital R** | 33, 33, -8 | 21 | 5.44 | 0.012 |
| **Frontal Inferior triangular R** | 52, 32, 18 | 51 | 5.98 | 0.004 |
| **Frontal Inferior triangular R** | 46, 18, 27 | 46 | 5.52 | 0.004 |
| **Frontal Lateral Orbital L** | -38, 54, -4 | 58 | 5.51 | 0.003 |
| **Frontal Medial L** | -42, 24, 33 | 23 | 5.32 | 0.011 |
| **Anterior cingulum L** | -8, 36, 22 | 116 | 5.41 | 0.001 |
| **Anterior cingulum L** | 10, 46, 16 | 18 | 5.19 | 0.014 |
| **Anterior cingulum L** | 12, 39, 0 | 15 | 5.22 | 0.016 |
| **Anterior cingulum R** | 6, 34, 26 | 72 | 5.62 | 0.001 |
| **Anterior cingulum R** | 4, 48, 24 | 31 | 5.47 | 0.008 |
| **Medial cingulum L** | -6, 18, 38 | 20 | 5.40 | 0.012 |
| **Medial cingulum R** | 6, 20, 36 | 25 | 5.17 | 0.010 |
| **Rectus R** | 6, 34, -16 | 72 | 5.62 | 0.002 |
| **Inferior Temporal R** | 48, -4, -36 | 114 | 5.50 | 0.001 |
| **Inferior Temporal R** | 56, -18, -27 | 35 | 5.24 | 0.007 |
